# The first record of the ectoparasite fly *Carnus hemapterus* for the Southern Hemisphere

**DOI:** 10.1016/j.ijppaw.2026.101194

**Published:** 2026-01-17

**Authors:** Paula Maiten Orozco-Valor, Luciano Damián Patitucci, José Hernán Sarasola

**Affiliations:** a Centro para el Estudio y Conservación de las Aves Rapaces en Argentina (CECARA), Facultad de Ciencias Exactas y Naturales, Universidad Nacional de La Pampa, Avda. Uruguay 151, Santa Rosa, La Pampa, 6300, Argentina; b Instituto de las Ciencias de la Tierra y Ambientales de La Pampa (INCITAP), Consejo Nacional de Investigaciones Científicas y Técnicas (CONICET), Calle Rivadavia 236, Santa Rosa, La Pampa, 6300, Argentina; c Museo Argentino de Ciencias Naturales “Bernardino Rivadavia”, Consejo Nacional de Investigaciones Científicas y Técnicas (CONICET), Av. Ángel Gallardo 470, C1405DJ, Ciudad de Buenos Aires, Argentina

**Keywords:** Argentina, Birds of prey, Hematophagous fly, Nestlings, American kestrel, Chimango caracara

## Abstract

The genus *Carnus* (family Carnidae, Order Diptera) comprises five species of small-bodied and blood-sucking flies that parasitize nestlings of wild bird species. Almost all species in this genus are restrictely distributed across different continents in the Northern Hemisphere, particularly *Carnus hemapterus*, which is a widespread ectoparasite of many bird species. Here, we report *C. hemapterus* parasitizing wild birds in central Argentina, resulting in the first record for the species in the entire Southern Hemisphere. Individual flies of *C. hemapterus* were found in nestlings of two bird of prey species, the American kestrel, already recorded as a host of *C. hemapterus* in North America, and the Chimango caracara, a new host species for this ectoparasite. Consistent with the species’ life cycle, flies were observed only in nestlings but not in adult individuals captured in the same breeding areas. Besides the plausible reasons that could explain this new report, it significantly updates the global distribution for this ectoparasite taxon. Therefore, this record should draw the attention to ornithologists and parasitologists from large regions of the global South, which include some of the most important avian biodiversity host-spots, to this new ectoparasite-host interaction, which may affect a significant number of bird species and warrants investigation of its physiological and ecological impacts. Furthermore, beyond its role as an avian ectoparasite, *C*. *hemapterus* is involved in complex interespecific interactions, serving as a host for parasitoids and participating in multitrophic food webs within bird nests, which clearly warrant further research.

## Highlights

• First record of bird ectoparasite fly, *Carnus hemapterus* in the entire Southern Hemisphere.
• *C. hemapterus* were found parasitizing nestlings of wild birds of prey in Argentina.
• The American kestrel has already been recorded as a host in North America.
• The Chimango caracara represents a new host species for this ectoparasite fly.
• The presence of this fly can be underestimated in wild birds below the equator.

## Graphical abstract

**Figure:**
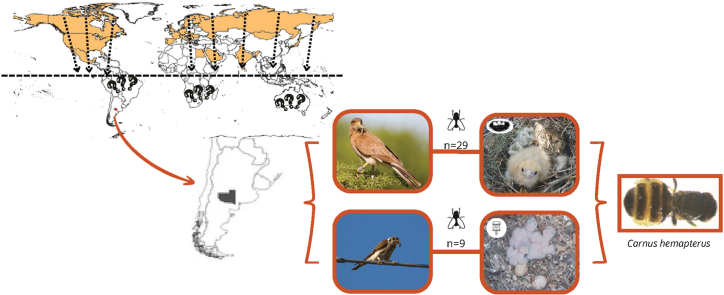

## 1 Introduction

Wild bird species can hold a large biodiversity of ectoparasites (Boyd, 1951), including flies of the family Carnidae (Swann and Stuke, 2021). This small family of the Diptera order comprises five genera with 92 described species (Courtney et al., 2017) of small-bodied (1–2 mm) flies with black-colored body parts. Several species in this family have saprophagous habits and are associated with carrion or feces. The genus *Carnus* in this family, however, comprises five species with hematophagous habits (Brake, 2011), all of which parasitize wild birds during the earliest birds' ontogenic phase at the nest.

Adults and larvae of these species can be found in both cavity and open-air bird nests (Brake, 2011; Grimaldi, 1997). The life cycle of *Carnus* fly involves mature individuals dispersing to a suitable host nest where they shed their wings and begin to feed on the blood of nestlings, usually selecting the bare skin areas of the under-wing axillary part of the host (Grimaldi, 1997; Veiga et al., 2019). Once established in the new host, adults lay their eggs, with larval development occurring on organic detritus at the nests. Newly emerged adult flies are fully winged, capable of dispersing again to a new host nest.

**Fig. 1:**
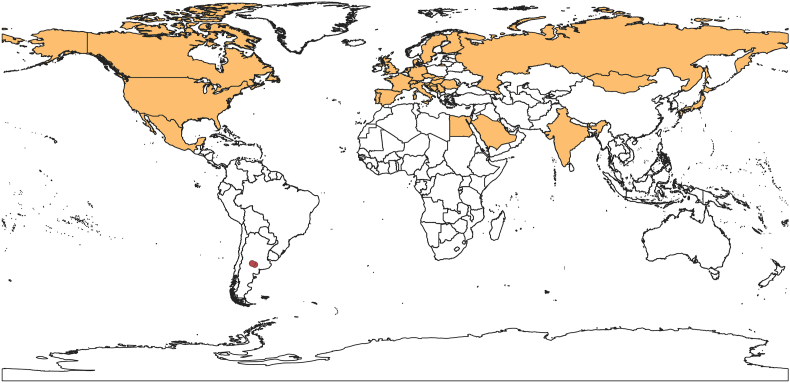
Geographic distribution of *Carnus hemapterus* in the world indicating the countries whithin the species ranges in the Northern Hemisphere (orange) and the location of the first record for the species in the Southern Hemisphere (red circle).

Among this genus of blood-sucking flies, *Carnus hemapterus* is probably the most widespread one. It is associated as a parasite of nestlings of at least 52 avian species (Grosso-Silva and Soeiro, 2021), and as for the entire Carnide family, their avian host are restricted to wild birds. Although Carnidae presents seemingly no economic impact, the ecological effects of the parasite behavior of *Carnus* on these wild host species are still uncertain (Swann and Stuke, 2021). Despite the wide taxonomic representation of bird hosts, *C. hemapterus* has a Holarctic-restricted distribution, as they are found only in the Northern Hemisphere (Brake, 2011; El-Hawagry et al., 2016; Ganbold et al., 2020; Grosso-Silva and Soeiro, 2021), spanning Europe, North America, Asia, and northern Africa (Fig. 1).

Here, we report *C. hemapterus* flies parasitizing birds for the first time in the Southern Hemisphere. Specifically, we report *C. hemapterus* as parasites of nestlings of two raptor species in central Argentina: the American kestrel (*Falco sparverius)* and the Chimango caracara (*Daptrius chimango*), belonging to the Falconidae family. From an entomological and taxonomic view, this report comprises a significant update to the global distribution of this genus of Diptera and for *C. hemapterus* in particular. Furthermore, this report should bring attention to ornithologists and parasitologists from the Southern Hemisphere on this newly described interaction between wild birds and this hematophagous ectoparasite species. New interactions include not only ecological and physiological implications for host birds but as those interspecific established by this fly species itself.

## 2 Materials & methods

### 2.1 Study area and sampling

Samples of ectoparasites were collected opportunistically during the 2023 austral spring and summer in La Pampa province, central Argentina, as part of a breeding population monitoring of American kestrels and Chimango caracaras in this region.

In the case of the cavity-nesting American kestrel, monitoring of breeding populations included regular visits to nest boxes set in a wide range of contrasting habitats, from agricultural lands near to Santa Rosa city (36°10′ S, 64°9′ O), to natural forest habitats dominated by Caldén (*Neltuma caldenia*) in the Parque Luro Natural Reserve, (36°55′ S, 64°16′W;Orozco Valor and Grande, 2020).

For the Chimango caracaras, however, open-to-air nests for the single breeding population of this semi-colonial raptor species were located in suburban areas near Santa Rosa and Toay cities (36°43′S, 64°16′W; Gallego-García et al., 2024).

In addition to nest monitoring, we also capture adult individuals of both raptor species at each of the breeding areas. Chimango caracaras were captured using walk-in traps baited with bones and meat (Solaro and Sarasola, 2017), while adult kestrels were captured inside the nest boxes while they were incubating. Nestlings and adult birds were banded, blood sampled, and measured before returning to the nest or released in the case of free-living adults. Before that, birds were externally examined for the presence of ectoparasites, including flies, mites, and other common blood-sucking ectoparasites previously reported for raptors in this region (Liébana et al., 2011; Orozco-Valor et al., 2019; Santillán et al., 2009, 2015). All ectoparasites collected were stored in Eppendorf vials with 90 % ethanol for later identification.

### 2.2 Specimens identification

The collected flies were identified using taxonomic keys and descriptions provided by Grimaldi (1997). To study the morphology of the terminalia, the terminal segments of selected specimens were detached and transferred to 90 % lactic acid at room temperature for two weeks. After neutralization with a 20 % acetic acid wash and two EtOH washes (70 % and 95 %), the genital structures were removed and temporarily mounted on concave glass slides in glycerine (O'Hara, 2021). After the study, the dissected parts were placed in a plastic microvial with glycerin and pinned with the respective specimen.

Photographs were taken using an Olympus DP 25 digital camera mounted on an Olympus SZX 16 stereomicroscope, and a Brunel digital camera mounted on a Motic optical microscope. Images were processed with the Olympus cellSens Standard software and Combine ZM. Scanning Electron Microscopy (SEM) images were taken with a Zeiss GeminiSEM 360 scanning electron microscope at the Museo Argentino de Ciencias Naturales “Bernardino Rivadavia”. The structures were dehydrated using 80, 90, and 99.5 % ethanol and coated with gold-palladium in a Thermo VG Scientific SC 7620 sputter coater. Voucher specimens were pinned and deposited in the Centro para el Estudio y Conservación de las Aves Rapaces en Argentina (CECARA), CONICET- Facultad de Cs. Exactas y Naturales, Universidad Nacional de La Pampa, La Pampa, Argentina and Museo Argentino de Ciencias Naturales “Bernardino Rivadavia” (MACN-CONICET), Buenos Aires, Argentina: MACN-En.

## 3 Results

We collected a total of 38 ectoparasite flies. In the case of American kestrels, the nine carnid flies were found in four different nests boxes and collected from one nestling of each brood. In addition, we collect 29 flies from Chimango caracaras nestlings in 13 nest: six nests with a single nestling parasitized and seven nests in which all brood members were infested.We did not observe any ectoparasite flies in adult American kestrels (12 individuals) and Chimango caracaras (61 individuals) captured at the breeding sites.

**Fig. 2:**
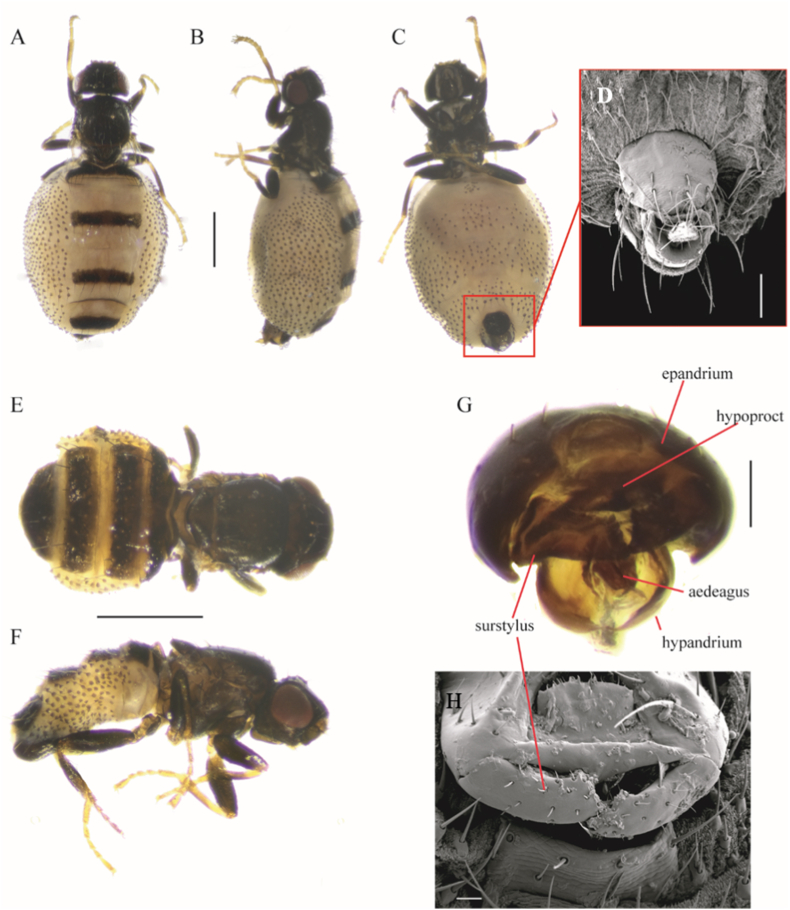
*Carnus hemapterus*. Female, (A) dorsal view, (B) lateral view, (C) ventral view (scale bar = 0.5 mm), (D) sternite 6 detailed (SEM) (scale bar = 0.1 mm). Male, (E) dorsal view, (F) lateral view (scale bar = 0.5 mm), (G) terminalia (scale bar = 0.1 mm), (H) surstylus (SEM) (scale bar = 0.02 mm).

All flies were identified following Grimaldi (1997) as adult individuals of *C. hemapterus*. Adult males and females of *Carnus* have dehiscent wings, notum with one pair of dorsocentral setae, and pleural membrane of the abdomen with dense setiferous spots. Abdominal sternites are lacking in females of *C. hemapterus*, while they are reduced in male individuals. Finally, we compared the shape of the surstylus to confirm the identity of *C. hemapterus* (Fig. 2) for all the individuals we collected.

## 4 Discussion

Here, we report individuals of *C*. *hemapterus* parasitizing nestlings of two raptor species in central Argentina, resulting in the first-ever report of this bird parasite fly for the entire Southern Hemisphere (Fig. 1). Furthermore, this report also comprises the second one for a fly of the *Carnus* genus for this whole hemisphere, since the only previous report of a *Carnus* sp. specimen belongs to central Africa (Grimaldi, 1997).The occurrence of *C. hemapterus* in nestling individuals at the nest but not in free-living adult birds was also consistent with the parasitizing behavior and life cycle of *C. hemapterus*.

Additionally, although *C*. *hemapterus* is a common parasite for a great diversity of wild birds, including nestlings of American kestrels in the USA and Canada (Dawson and Bortolotti, 1997; Swann and Stuke, 2021), this report also adds the Chimango caracara as a new host for this ectoparasite fly.

This singular record could be partly explained by inadequate surveys on avian ectoparasites for a large portion of the planet, including South Africa and Asia, Oceania, and particularly southern South America, or by a recent and previously undetected expansion of the range of this fly species that resulted in this record in central Argentina.

However, and although the ectoparasite community of wild raptor species in South America may remain poorly studied, there is a strong and long-standing tradition of ornithological research in this region on a wide range of bird taxa, including studies on avian ectoparasites. For Paraguay, for example, a recent review of avian research patterns over the last 25 years reveals that nearly 5 % of ornithological studies on birds (n = 203) focused on avian host ectoparasites (Esquivel et al., 2023). In Argentina, ectoparasite flies of the genus *Philornis* (Diptera: Muscidae) have been recorded in nestlings of more than 80 different species of wild birds in that country (Salvador and Bodrati, 2013). Moreover, inspection of the ectoparasite community in more than 850 individuals of burrowing owls, *Athene cunicularia*, in Argentina did not register the presence of *Carnu*s flies (Sáez-Ventura et al., 2022). Therefore, despite the number of studies on avian ectoparasites in the region, the presence of this fly species may remained unnoticed possibly due to low prevalence, nestling developmental stage at the time of birds sampling, wich relate with survey designs more proper for ornithological research than for ectoparsites studies, or a combination of several of these factors.

The phenology of parasitizing may also be taken into account as another reason for the lack of previous records of *Carnus* flies. If bird nestlings are examined as fledglings and late during the stage (i.e., close to the moment of leaving the nest), the life cycle of flies may be completed and in diapause (Václav et al., 2008; Valera et al., 2006), since adult flies reach a peak during early nestling stages and declining sharply as nestlings become fully feathered, (Václav et al., 2008), resulting in underdiagnosis of parasitization by the *Carnus* fly. Also, *Carnus* species present restricted habitats in close association with nests ditritus, making the specimens rarely collected by entomologists (Wheeler, 2010).

On the other hand, a recent and fast expansion of the species to southern latitudes may be unlikely. In birds, migratory species are highly capable of transporting ectoparasites over large distances in short time periods; later, after traveling with migratory bird hosts, ectoparasites may then transmit to resident birds. Long-distance juvenile dispersal movements, like those reported in our study area for both American kestrels (Orozco-Valor et al., 2022) and Chimango caracaras (Solaro and Sarasola, 2018), may also serve as dispersal processes for ectoparasites at a local or regional scale. However, in the case of *C. hemapterus*, individuals spend more of their lives either parasitizing nestlings or in the nest substrate and are absent in free-living individuals, although passive dispersal of ectoparasite eggs on the legs and plumage of adult birds cannot be totally ruled out.

This record of *C. hemapturus* in central Argentina, should bring attention to ornithologists and parasitologists from large regions of the global South, which include some of the most important avian biodiversity host-spots, about a new potential ectoparasite-host interaction for a significant number of bird species that needs to be investigated in terms of its physiological and ecological impact on the individuals. The presence of these flies on these two raptor species could also be related to the presence of carrion in the nest, where the larval stage could develop (Grimaldi, 1997). Beyond its role as an avian ectoparasite, *C*.*hemapterus* is involved in complex interespecific interactions, acting as a host for parasitoids and participating in multitrophic food webs within bird nests (Salido et al., 2021; Struwe et al., 2023) which clearly warrant further research.

## 5 Conclusions

Our findings broaden the current distribution of this parasite fly and of the *Carnus* genus as a whole in the southern part of the globe. Additionally, it supports the call to ornithologists to address the gap on ectoparasites in wild birds in the southernmost areas, including other locations with a longer ornithological research history. Further research is needed on the presence of this parasite on wild birds and its potential ecological effects, which nowadays is mainly based on the Northern Hemisphere. Our findings may suggest that the presence of these hematophagous flies can be underestimated in wild birds in other locations below the equator and particularly for other bird species in central Argentina. Given the close relation of this particular fly to avian hosts, we encourage the ornithologist community to inspect wild birds for this ectoparasite.

## CRediT authorship contribution statement

**Paula Maiten Orozco-Valor:** Writing – review & editing, Writing – original draft, Visualization, Validation, Methodology, Conceptualization. **Luciano Damián Patitucci:** Writing – review & editing, Writing – original draft, Visualization, Validation, Methodology, Investigation, Data curation, Conceptualization. **José Hernán Sarasola:** Writing – review & editing, Writing – original draft, Visualization, Validation, Methodology, Conceptualization.

## Ethical approval

Fieldwork and all field procedures were conducted under permits from the Subsecretaría de Ecología (La Pampa province, Argentina) to work in Parque Luro Natural Reserve and from the Dirección de Recursos Naturales (La Pampa province, Argentina) in the remaining study areas.

## Funding

Financial support was partially provided by FONCyT and the UNLPam (PICT-2020-SERIEA-00590), and by the PI-41 of the Department of Biological Sciences, National University of La Pampa, FECyN- UNLPam. Hawk Mountain Sanctuary (Project Soar), Idea Wild.

## Declaration of competing interest

The authors declare that they have no known competing financial interests or personal relationships that could have appeared to influence the work reported in this paper.
